# Fiberoptic array for multiple channel infrared neural stimulation of the brain

**DOI:** 10.1117/1.NPh.8.2.025005

**Published:** 2021-04-22

**Authors:** Mykyta M. Chernov, Robert M. Friedman, Anna W. Roe

**Affiliations:** Oregon Health and Science University, Oregon National Primate Research Center, Division of Neuroscience, Beaverton, Oregon, United States

**Keywords:** infrared neural stimulation, intrinsic optical imaging, non-human primate

## Abstract

**Significance:** We present a new optical method for modulating cortical activity in multiple locations and across multiple time points with high spatial and temporal precision. Our method uses infrared light and does not require dyes or transgenic modifications. It is compatible with a number of other stimulation and recording techniques.

**Aim:** Infrared neural stimulation (INS) has been largely confined to single point stimuli. In this study, we expand upon this approach and develop a rapidly switched fiber array capable of generation of stimulus patterns. Our prototype is capable of stimulating at nine separate locations but is easily scalable.

**Approach:** Our device is made of commercially available components: a solid-state infrared laser, a piezoelectric fiber coupled optical switch, and 200-μm diameter optical fibers. We validate it using intrinsic optical signal imaging of INS responses in macaque and squirrel monkey sensory cortical areas.

**Results:** We demonstrate that our switched array can consistently generate responses in primate cortex, consistent with earlier single channel INS investigations.

**Conclusions:** Our device can successfully target the cortical surface, either at one specific region or multiple points spread out across different areas. It is compatible with a host of other imaging and stimulation modalities.

## 1 Introduction

Over the past two decades, there has been considerable interest in the development of optical methods to supplement traditional electrophysiological techniques in brain machine interfaces and for basic science applications.^1^ For example, optogenetics has revolutionized neuroscience by allowing one to target specific neuronal populations,^2^ whereas genetically expressed calcium sensors enable one to study circuit dynamics *in vivo* at the level of a single cell.^3^^,^^4^ One major development has been the use of patterned illumination to target multiple regions across the brain, opening up the possibility to interact dynamically with the flow of information rather than target a single point or brain region.^5^ In this paper, we extend the possibility of multi-spot stimulation to another optical technique: infrared neuronal stimulation (INS).^6^ INS relies on the use of brief pulses of infrared light to modulate neural activity via thermally induced changes in membrane capacitance, which in turn produce action potentials.^7^^,^^8^ Additional modulatory effects due to a change in ion channel properties have been hypothesized as well.^9^ The chosen wavelength, near one of the absorption peaks of water (e.g., 1.875 or 2.12  μm), is usually delivered via optical fibers. The combination of high absorption (path length on the order of a few hundred micrometers), short pulses (∼1  ms), and small spot sizes (∼100  μm) create a region of thermal confinement that allows the stimulus to vary in magnitude while remaining highly focal. Another useful feature of INS is its compatibility with many other modalities including MRI and intrinsic optical imaging.^10^ Unlike optogenetics, it does not rely on genetic modification of tissue, making it attractive for a number of potential applications in human patients, including cochlear implants,^11^ brain machine interfaces, and cardiac pacemakers.^12^

### 1.1 Design Rationale

Previously, patterned optical stimulation has been achieved with optogenetics^5^ and caged neurotransmitter release. The light sources were either multiple sources (usually arrays of LEDs) or raster scanning using galvo mirrors, digital micromirror devices (DMDs), or liquid crystal spatial light modulation arrays (SLMs), which use electrically sensitive liquid crystals to alter the refractive index of the waveplate and create interference patterns, both in two- and three-dimensions.^13^ However, application of these techniques is challenging with INS for three reasons: (1) high peak power required for INS (on the order of 1 W versus a few mW for optogenetics) to deposit enough radiant energy within a sub-millisecond pulse. (2) Solid-state laser stacks, the most cost-effective light sources for INS, are multimode lasers with low beam quality and complex polarization. These factors make collimation and free beam manipulation difficult. (3) The low transmittance of glass optics at the absorption peak of water around 2  μm used in INS and the expense of IR optics. Most SLMs are not capable of handling the energies required and are inherently inefficient for patterns that do not fill a large fraction of the mirror surface at any given time. A galvo mirror setup has been used with a relatively low power (400 mW) single mode INS light source for embryonic heart pacing.^14^ The 1445-nm wavelength light used in that experiment, however, is not as strongly absorbed, creates less localized stimuli and generates more non-specific thermal effects. Due to the difficulty in maintaining a well-collimated beam and the expense of IR optics, we decided that a switched fiber array solution would be the easiest and most cost effective. We chose a piezoelectric switch (Piezosystem Jena, Jena, Germany) because it is able to handle the necessary radiant exposures on the order of $1\;{J/\mathrm{cm}}^{2}$, has <20% optical power losses, and has switching times of about 3 ms. Competitive technologies are either too slow (stepper motor-based switches) or cannot handle the optical power (DMD-based switches). The proof of concept unit has a capacity of nine channels but with the low power losses observed 81 channels could easily be achieved by adding a second stage of nine identical switches. The tip of the array contacting the brain is a linear array of 200  μm diameter optical fibers with no spacing in between, which gives a spacing of 240  μm from one fiber center to the other (accounting for the cladding). A detailed schematic of our design is shown in Fig. 1 of the Supplementary Material.

### 1.2 Device Validation

We validated our device in an anesthetized primate model. Our lab’s expertise lies in the study of functional domains in primate sensory cortex.^15^^,^^16^ These are submillimeter-sized regions of the brain that are easily identified with intrinsic optical imaging, a method that relies on reflectance of deoxygenated blood under red light illumination to gauge neuronal activity-related changes in tissue metabolism.^17^ The spatial selectivity of INS makes it possible to stimulate single functional domains in both anesthetized and awake behaving animals.^15^ Previously, we have shown that INS can drive changes in intrinsic signal in both rodents and primates,^18^^,^^19^ modulate neuronal activity measured with electrophysiology,^19^ and affect animal behavior.^20^ Here, in a proof of concept study, we extend the capability of INS from targeting single points to generating moving patterns with a fiber array.

Targeting multiple cortical areas in precisely controlled temporal patterns of stimulation will provide the capability to (1) investigate how spatial distinct nodes in neural networks integrate spatial and temporal information, and (2) create dynamic and complex percepts by rapidly switching stimulation locations. Our device, with a 200-μm spot size and several millisecond switching speed (roughly the duration of an action potential), is capable of achieving these objectives in studies of cerebral cortex.

## 2 Methods

All experiments were approved by the institutional Animal Care and Use Committee at Oregon Health and Science University. For a detailed description of INS and intrinsic optical signal imaging, the reader is referred to earlier publications.^16^^,^^21^

### 2.1 Surgical Procedure

Experiments were carried out in visual cortex of two rhesus macaques (Macaca mulatta) and somatosensory cortex of one squirrel monkey (Saimiri sciureus). Animals were anesthetized with Ketamine (10  mg/kg), intubated and placed in a custom-made stereotaxic frame. Analgesia was ensured by injection of buprenorphine (0.03  mg/Kg) in the squirrel monkey and hydromorphone (0.1  mg/kg) in the macaques. Additional analgesia in the macaque was provided by continuous rate infusion (CRI) of ketamine (0.2  mg/kg/h). Surgical procedures were carried out under isoflurane (1% to 2%) in oxygen, and data were collected under propofol anesthesia (3  mg/kg induction dose and 12  mg/kg/h CRI) in macaques and isoflurane (1% to 2%) in the squirrel monkey. The animal’s body temperature was maintained at physiological levels using a recirculating water blanket. Additional physiological monitoring included heart rate, oxygen saturation, blood pressure, and expired (or end tidal) ${\mathrm{CO}}_{2}$. Animals were maintained on a ventilator to ensure proper oxygenation. Under aseptic conditions, an incision was made along the midline of the scalp and a 1 to 2 cm diameter craniotomy was performed using a rotary tool with a dental drill bit. Bone was removed, and the dura was carefully lifted and resected using a pair of microdissection scissors. A 0.2-mm-thick quartz coverslip was placed on the surface of the exposed brain and secured in place with 4% agar. After the completion of the experiment, the animals were either euthanized with a barbiturate (Beuthanasia, 1 to 2 ml) and sent to necropsy for tissue bank harvesting or recovered by tucking a piece of artificial dura (Tecoflex, Lubrizol Inc. Wickliffe) under the dural margins, replacing the previously removed bone flap, securing it with dental acrylic, suturing the skin and finally reducing the isoflurane concentration to recover the animal from anesthesia. Post-surgical care included administration of antibiotics (Cefazolin 25  mg/kg IV) and Buprenex (0.03 to 0.06  mg/ml) twice daily for three days.

### 2.2 Infrared Neural Stimulation

The cortical surface was stimulated using an array of 200-μm-diameter multimode silica optical fibers (Thorlabs, Newton New Jersey) placed against a cranial optical window (quartz cover slip). The 200-μm size was a compromise between the desire for high spatial resolution and the ability to collimate a laser diode stack’s output. The fibers of the array formed a 60-deg angle with the surface of the cortex. This position was chosen due to the limited space between the cortical surface and the imaging lens and caused an ellipsoidal, rather than circular illumination spot, with about a 16% difference between the long and short axes lengths. The array was connected to a nine-channel piezoelectric optical switch (Piezosystem Jena, Jena, Germany) coupled to an 1875-nm center wavelength 6-W diode laser via a subminiature version A (SMA 905) connector (Laserglow, Toronto, Canada). Previous studies demonstrated that pulses of 0.3 to $0.7\;{J/\mathrm{cm}}^{2}$ were effective in eliciting intrinsic optical signals and electrophysiological responses. Pulses of 0.25 ms in duration were delivered in 0.25 to 0.5 s long trains using TTL pulses originating from an Arduino-based (Arduino, Somerville Manchester) custom-built programmable signal generator. The same device was used to control the fiber optic switch.

Power output was confirmed using a Thorlabs power meter (PM100d Newton New Jersey) with a Thorlabs power head (S302c). Optical pulse waveforms were monitored using an InGaAs photodiode connected to a custom built transimpedance amplifier circuit^22^ connected to a digital storage oscilloscope (TDS 2002B, Tektronix, Beaverton, Oregon). For calculations of radiant energy delivered per pulse, we assumed the beam profile to be top hat.^23^ Average power measured by the power meter was divided by the duty cycle to obtain the peak power, which was in turn multiplied by the pulse width to obtain the amount of energy deposited per pulse. The final result was given as energy divided by the area of the fiber core in $J/{\mathrm{cm}}^{2}$.

### 2.3 Intrinsic optical signal imaging and data analysis

The cortical surface was illuminated with an LED at a center wavelength of ∼632  nm. Images were acquired at 4 Hz using a cooled CCD camera connected to a purpose-built data acquisition system (Optical Imaging Inc, Rehovot, Israel). Both sequences including INS and blank sequences were collected in random order in blocks of 20 to 40 trials. To avoid residual signal from contaminating temporally adjacent trials, we chose a minimum interstimulus interval of 6 s. Intrinsic signal data frames were analyzed using custom MATLAB scripts. To examine signal change from baseline, the first frame of each condition, collected prior to INS stimulation, was subtracted from the rest of the image sequence. Conditions from all trials (>30/condition) were averaged to increase signal to noise ratio. To remove large reflectance changes due to vascular artifacts, pixels with absolute values >1 standard deviation from the median were excluded. Images were filtered with a Gaussian high-pass filter (5-pixel kernel 0.1 mm) and a median low-pass filter (2-mm kernel). Pixels with statistically significant reflectance changes were determined using T-test (p<0.001).

## 3 Results

### 3.1 Array Construction and Benchtop Testing

**Fig. 1:**
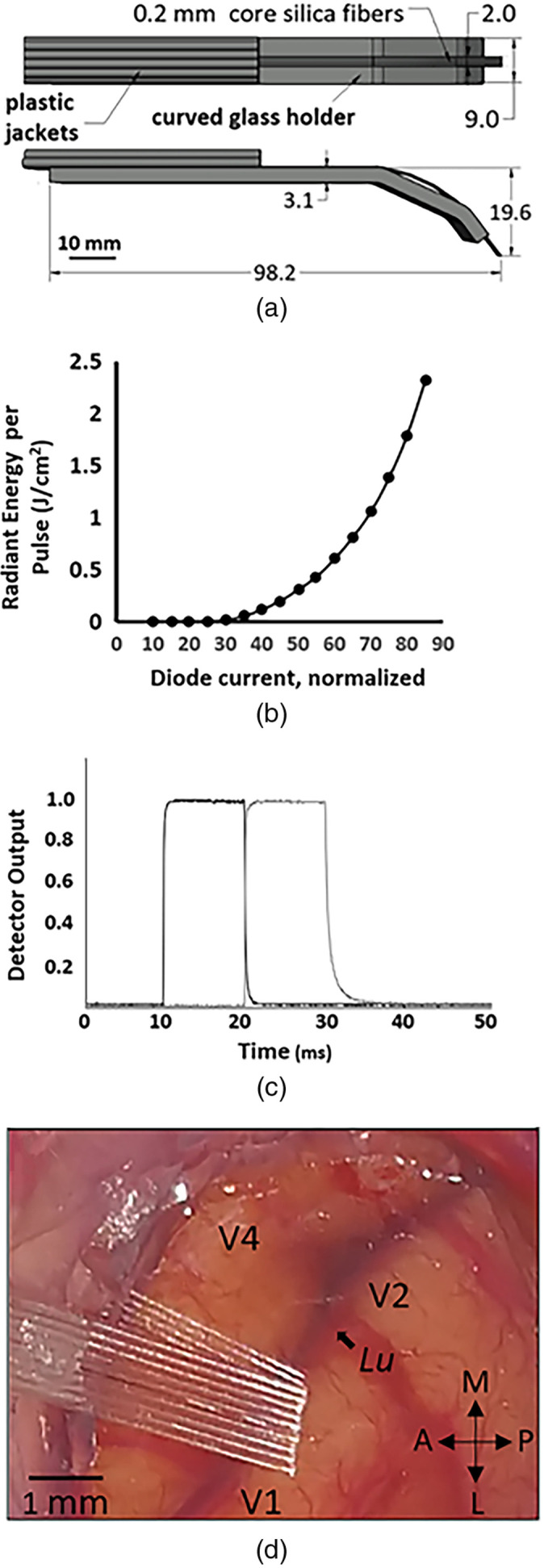
(a) A linear array of nine 200-μm diameter core silica fibers (240-μm total diameter) glued to a glass holder. (b) Irradiance of the diode infrared laser (λ=1870  nm) used in the experiments. The laser current value reading was available as a digital readout on the laser provided by the manufacturer and the irradiance was derived from measurement of optical power using a power meter as described in the methods. (c) Light detected from two adjacent fibers switched one after another, confirming that the process takes place within the value specified by the manufacturer (3 ms). The measurements were obtained using an amplified InGaAS photodiode with the gain adjusted to ensure that the device did not saturate. (d) The nine channel array placed over a 200-μm-thick quartz coverslip for stimulation of monkey visual cortex. The lunate sulcus (lu) and V1, V2, and V4 identify the cortical regions in the craniotomy. A, M, L, and P designate the anterior, medial, lateral, and posterior orientations of the brain.

While other layouts and fiber diameters are possible, we constructed a 1×5 and a 1×9 linear array of 200-μm diameter fibers directly apposed to each other [Fig. 1(a)]. The fibers were secured against a small piece of a glass coverslip with glue to ensure that they laid flat relative to each other. The coverslip was then glued to the tip of a larger curved glass holder and the fibers were gently curved back and secured with epoxy. The array was connected to a diode laser with a center wavelength of 1870 nm (Laserglow, Toronto, Canada) to deliver 250-μs pulses in 200-Hz trains lasting between 250 and 500 ms, parameters that were previously found to be effective for eliciting intrinsic optical imaging, MRI, and electrophysiological responses in the cortex of both rodents and primates.^10^^,^^18^^,^^19^ The magnitude of the INS stimulus was changed by varying the laser diode current to generate between 0.4 to $0.7\;{J/\mathrm{cm}}^{2}$ per pulse [Fig 1(b)]. Given that the power losses within the 1×9 fiber switch and the fiberoptic connectors (SMA 905 type) were 20%±4% for each channel, the laser diode needed to operate at around 60% of its maximum current to deposit 0.4 to $0.7\;{J/\mathrm{cm}}^{2}$ per pulse [Fig 1(b)]. The switching speed was confirmed to be within the 3 ms specified by the manufacturer [Fig. 1(c)], which is fast enough for most electrophysiological studies.

### 3.2 In Vivo Testing

**Fig. 2:**
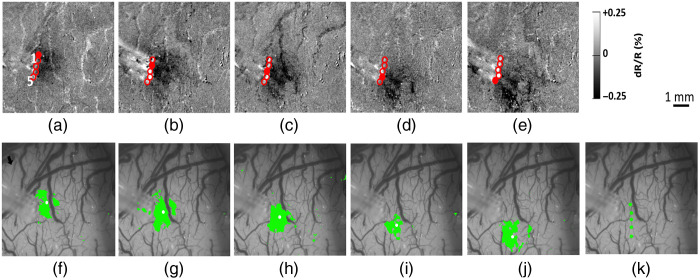
(a)–(e) Stimulation through an array of five adjacent optical fibers, moving from top to bottom of the image ($0.34\;{J/\mathrm{cm}}^{2}$ per pulse, 0.25 ms pulse width (PW), 200 Hz frequency and 0.5 s train duration). Fiber tips are circled in red, with the filled circle indicating the fiber stimulated for that image. (f)–(j) Areas of significant increase in intrinsic optical signal (student’s paired T-test versus no stimulation, p<0.001) following stimulation at locations in (a)–(e). Yellow dots identify the centroid of the activation. (k) Centroids in (f)–(j) shown all on the same figure.

The device was tested on three animals (visual cortex in two rhesus macaques and somatosensory cortex in one squirrel monkey) during four anesthetized imaging sessions. A typical craniotomy is shown on Fig. 1(d), where a size of about 10 to 20 mm in diameter affords access to multiple cortical areas. Since some areas are inevitably blocked from view by the array, a thinner five channel design was sometimes more expedient, especially for smaller craniotomies, and was used in the squirrel monkey (Fig. 2). We wanted to determine whether the array produced activations that were consistent with our previous experience using single fibers and whether the evoked signal magnitude was consistent across fibers. Figure 2 shows the responses to stimulation ($0.34\;{J/\mathrm{cm}}^{2}$) through a five-channel array, with equally sized activation spots moving from one end of the array to the other. The dark regions indicate increased intrinsic optical signal, which is correlated with increased metabolic activity. They appear darker because oxygen-poor blood is less reflective under red light. For the activations from the single fibers in Figs. 2(a)–2(e), the areas that showed significant activation based on the T-test (paired T-test against a no-stimulus control; p<0.001) are shown in green in Figs. 2(f)–2(j). While the regions of activation are broad (∼1  mm in size) they are approximately circular and their location is tightly correlated to the illumination spot, as shown by tracking the centroids of the activated regions, which were calculated using ImageJ software’s built-in analysis feature (National Institutes of Health, Bethesda, Maryland) by averaging all the x and y pixel values within the areas of activation Figs. 2(f)–2(k). The variability in centroid size was estimated by splitting the dataset used to calculate the T-test into two randomized sets of trials S1 and S2 and calculating the differences in the x and y coordinates between the two sets (S1x−S2x, S1y−S2y) for Figs. 2(f)–2(j). The differences between the centroid coordinates for each x and y pair were pooled. The average variation was 160  μm and the standard deviation was 180  μm (N=10, S1x−S2x, and S1y−S2y combined). The distance between the centroids is 270±27  μm (standard deviation), which is close to but slightly larger than the 240-μm distance between the fiber tip centers. This increase can be due to a number of factors, such as slight separation of the individual fiber tips as they contact the surface of the coverslip and the oblique orientation of the array with respect to the cortical surface. Given the ∼1-mm size of activated regions and 240-mm fiber separation, there was quite a bit of overlap in the activated regions following stimulation of adjacent fibers. Quantifying the amount of overlap by calculating the ratio of activation overlap (overlapped area/total area) confirmed there was less overlap with larger fiber separation. The ratio of overlap for pairs of fibers separated by 1, 2, and 3 fiber diameters decreasing from 0.4 (0.07 SD) to 0.19 (0.01) to 0.08 (0.02), respectively. Activation regions following stimulation through the fibers at the edges of the array (four fiber diameters apart) did not overlap.

**Fig. 3:**
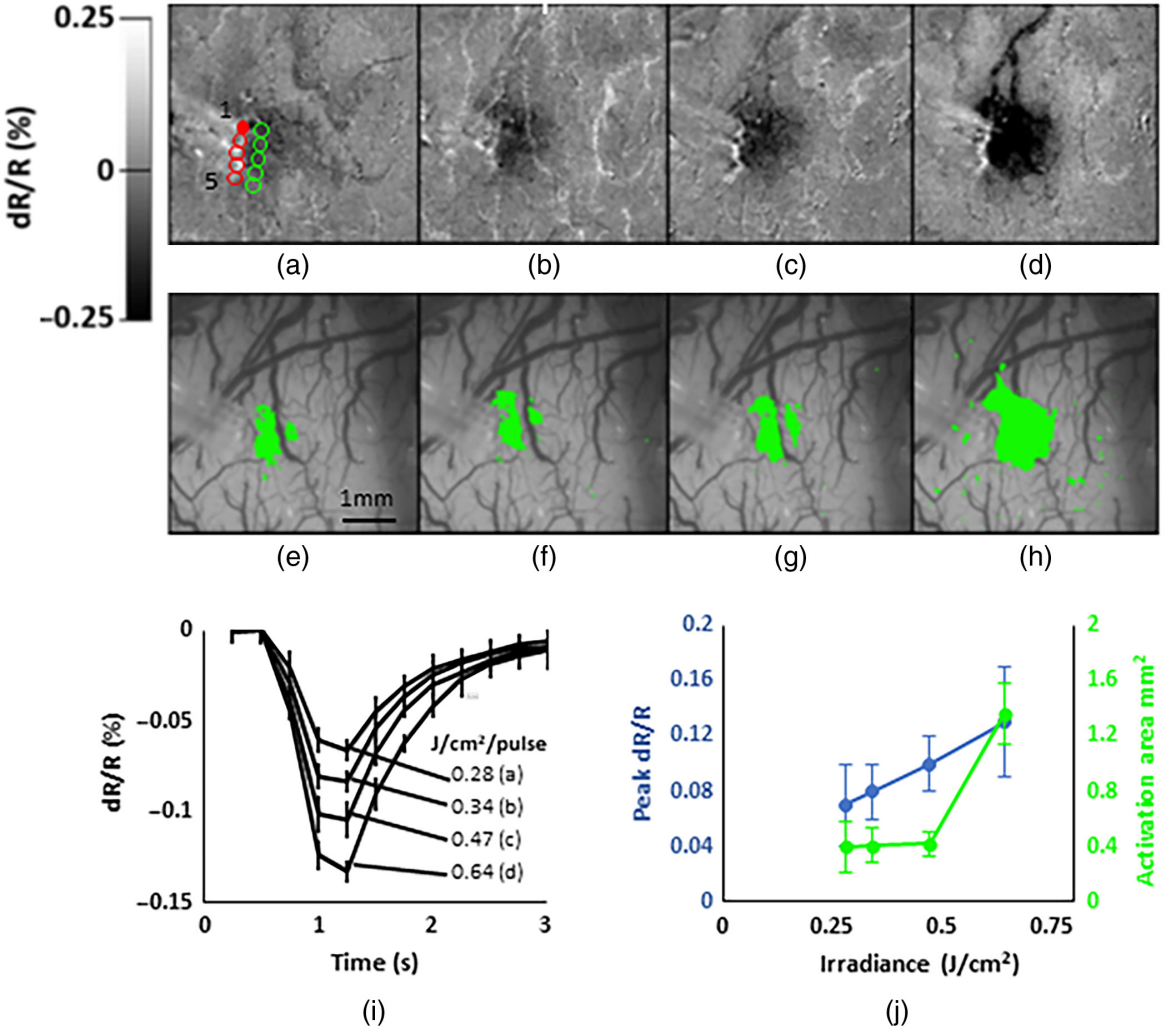
(a)–(d) Stimulation of fiber 1 [top filled red dot in (a) at different irradiances, left to right: 0.28, 0.34, 0.47, and $0.64\;{J/\mathrm{cm}}^{2}$ per pulse]. Stimulation parameters were 0.25-ms PW, frequency of 200 Hz and 0.5 s train duration. (e)–(h) Areas of significant activation [of (a)–(d)] compared to blank (p<0.001, paired T-test, quantified in green). (i) For the four different irradiances, time courses of activation averaged for the 5 fibers from regions of interest located immediately to the right of the stimulating fibers [hollow green circles in (a)]. Stimulation began at 0.5 s. (j) Plots of peak signal (blue curve) and significant area of activation (green curve) versus irradiance. Error bars are standard deviation.

Increasing the irradiance per pulse resulted in stronger signal. This is shown for one of the channels in Figs. 3(a)–3(d). The time courses of the changes in reflectance averaged for the five fibers for the four irradiance levels are shown in Fig. 3(i). Consistent with previous INS studies, the intrinsic signal takes about a second to peak and has a magnitude on the order of 0.1%.^19^ Signal amplitude (decrease in tissue reflectance) increased linearly as a function of radiant exposure [Fig. 3(j), blue line] and was fairly consistent between fibers as demonstrated by the standard deviation values. The area of significant activation (paired T-test against a no-stimulus control; p<0.001), on the other hand, remained at about $0.4\;{\mathrm{mm}}^{2}$ for the lower three radiant exposures but increased ∼3 times at the highest value of radiant exposure tested [Fig. 3(j), green line], probably due to nonspecific thermal effects. For the activations from the single fiber Figs. 3(a)–3(d), the areas that showed significant activation based on the T-test are shown in green in Figs. 3(e)–3(h).

### 3.3 “Moving Spot” Stimuli

**Fig. 4:**
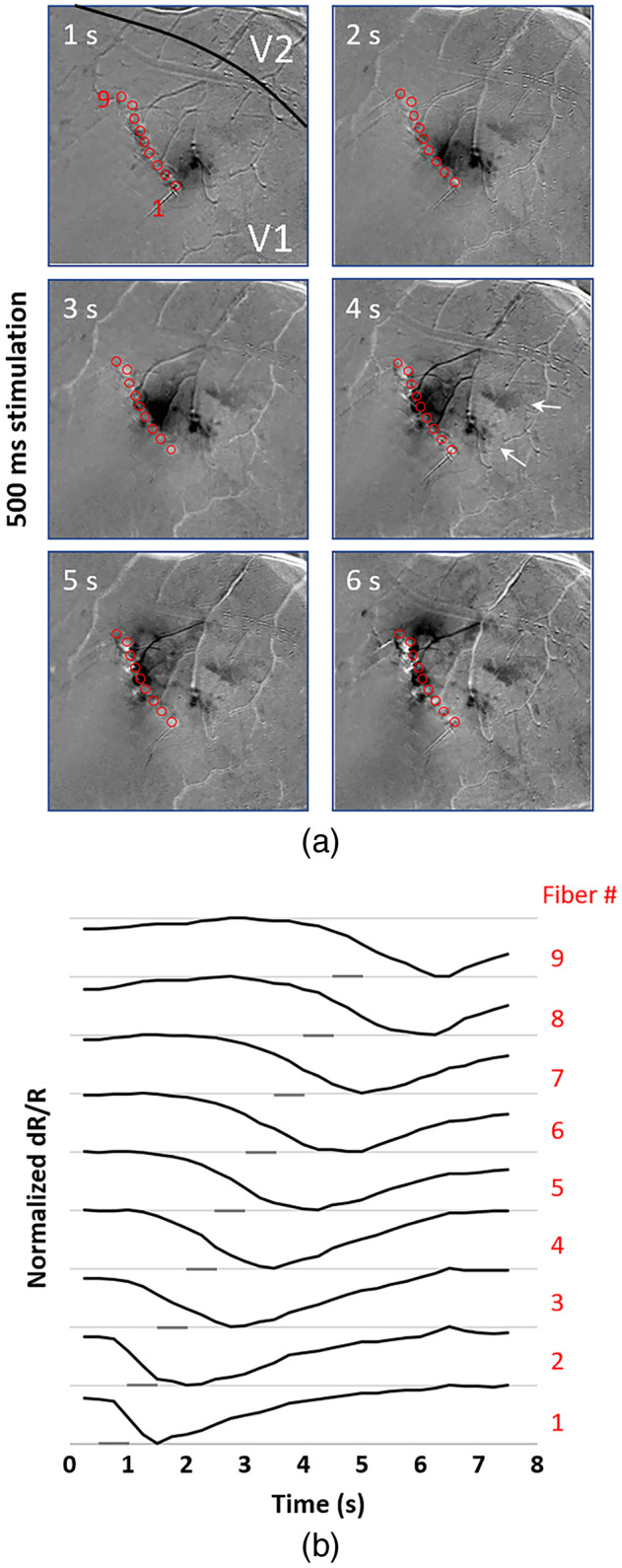
Intrinsic signal imaging of sequential stimulation of adjacent fibers of a nine channel linear array described in Fig. 1, generating a moving dot stimulus moving up and to the left of the image. (a) Series of images showing the activation from 1 to 6 s. Red circles are over fiber tips. Numbers in the corner of each image indicate seconds after start of imaging. Duration of the stimulation train is 0.5 s for each fiber. Irradiance is $0.7\;{J/\mathrm{cm}}^{2}$, pulse width is 0.25 ms, and frequency 200 Hz. (b) Time courses show the buildup of the signal over time after the start of stimulation at each illumination spot. ROIs are 250-μm diameter circles adjacent to the stimulating fiber. Gray bars indicate the time of the laser stimuli. White arrows point to possible secondary activation regions. Black line indicates approximate border between areas V1 and V2 based on intrinsic signal optical imaging. Videos of intrinsic imaging responses to INS applied using a linear nine channel array. (a) Array stimulation of adjacent points with pulse train duration of 250 ms (Video 1, mp4, 1306 KB, [URL: https://doi.org/10.1117/1.NPH.8.2.025005.1]).). (b) Array stimulation of adjacent points with pulse train duration of 0.5 s. Image frame rate 4 Hz, video duration 6 s (Video 2, mp4, 1551 KB [URL: https://doi.org/10.1117/1.NPH.8.2.025005.2]).). Field of view in both cases is 5 mm.

We previously conducted an experiment in which macaques were shown spots moving across their visual field in different directions. Using intrinsic imaging, motion-sensitive domains were identified in V2.^24^ This study was one of the motivating factors behind the design of a linear array, which would theoretically allow us to simulate motion percepts by sequential stimulation of retinotopically organized V1 cortex. To simulate different motion speeds, we used stimulation trains of either 250 or 500 ms at each location. We estimated the extent of the visual field in our craniotomy based on its mediolateral location to be ∼4 visual degrees. Given the size of the array (∼2-mm wide), and the time it took for the spot-by-spot stimulation to travel from one end to the other we were able to produce patterns corresponding to a slow-moving spot traversing the visual field at ∼0.25- to 0.5-deg per second. As soon as stimulation was done at one location, the next location was stimulated. The results for 500-ms stimulation are shown in Fig. 4 and Video 1 and Video 2 (both 250- and 500-ms long stimulation trains). In Fig. 4(a), in the series of image frames, the activation moves over time from near the bottom of the fiber array (fiber 1) toward the top (fiber 9) paralleling the order of stimulation. Shorter stimuli result in slightly lighter activation than the longer stimuli but the two stimulus durations do not show major differences in the activation patterns (Video 1 and Video 2). The intrinsic signal responses have a longer duration than the interval between consecutive stimuli therefore the resulting signal appears as a streak rather than a single spot moving along the array, with the trailing edge disappearing a few seconds after stimulation. As shown in Fig. 4(b), since the hemodynamic response takes about a second to reach its maximum, most of the activation appears to lag behind the currently stimulated channel. Although we did not observe activation in motion domains in V2, we did notice several areas of secondary activation in V1, which are highlighted with white arrows at the 4 second mark in Fig. 4(a). Our failure to detect motion domains in V2 was possibly due to the relatively slow movement of the stimuli along the cortical surface (0.4  deg/s) or to the step-like fiber stimulation pattern compared to the visually presented spot stimulus used in previous study (1.5 or 4.5  deg/s).^24^ This issue may be resolved in the future using shorter pulse trains or placing the fibers farther apart from each other.

## 4 Discussion

We have developed a device capable of delivering infrared neural stimuli to the cortical surface with millisecond temporal precision and a spatial resolution of a few hundred micrometers. While our prototype consists of nine channels, a higher channel count device could be easily constructed by cascading multiple units. We validated our device using intrinsic optical signal imaging, which we have used extensively in the past in combination with INS, optogenetic, or electrical stimulation to study cortical organization at the submillimeter level in rodents and non-human primates. In these studies, we explored the influence of stimulation on functional domains and their responses to visual or tactile stimuli. However, our studies were previously limited to only a single or a pair of points. The development of the INS array creates the opportunity not only to target multiple regions across the cortical surface, but also to generate dynamic stimulation patterns, which combined with sensory stimulus presentation, can be used to study how the flow of information is processed by cortex.

An INS optical array allows for flexibility in targeting neural activity of different structures that can be detected using electrophysiology or voltage sensitive dyes, or indirectly by measuring changes in blood flow using MRI or intrinsic signal optical imaging. The array elements can be concentrated in one cortical area or multiple areas may be targeted. The intrinsic sensitivity of the brain to IR light eliminates the need for genetic manipulation for stimulation or introduction of dyes for imaging. This is particularly important in the case of primate research where it is difficult to achieve dense transfection with viral constructions of large areas. Some promising approaches are on the horizon such as convection-enhanced vector delivery,^25^ intracerebroventricular,^26^ or even intravenous delivery,^27^ but these approaches are not without drawbacks.^28^^,^^29^ INS has also been used to study interareal connections in conjunction with functional magnetic resonance imaging, which opens up possibilities for study of both cortical and subcortical areas.^10^^,^^30^

Another advantage of INS is its ability to deliver spatially localized stimuli. Although confocal microscopy enables precise targeting of small structures, achieving irradiances sufficient for stimulation in well localized regions at the mesoscale has been a challenge in primate optogenetic studies due to light scattering.^31^ INS, on the other hand, is absorption-limited and can be spatially well-confined.^21^ The focal nature of INS makes it an attractive method for *in vivo* cortical tracing in conjunction with a variety of imaging techniques.^15^ For example, we have used optical imaging to demonstrate that targeting single ocular dominance columns in the primate visual cortex produced stronger activation than simultaneous stimulation of adjacent columns with a large fiber, which we believe is due to binocular rivalry.^19^ INS has also been used to study interareal connections in conjunction with functional magnetic resonance imaging.^10^ A switched multi-channel stimulator described here is the next logical step for further use of INS in functional tracing and opens up several interesting possibilities. One possibility is the creation of simple stimulation patterns by interleaving the stimulation trains among several fibers arranged in a specific shape. For example, one could target a series of the same eye ocular dominance columns with a linear array. Another research direction would be to generate inverse models of visual stimuli based on V1 representations obtained with fast optical imaging such as voltage sensitive dyes.^32^ The next step would be to create complex visual percepts using an INS array to generate a stimulation pattern in V1.

### 4.1 Limitations and Future Directions

The current prototype relies on piezoelectric switching between the light source and output channels. It has only a few off the shelf components. However, it is not able to deliver truly simultaneous stimulation to multiple locations—only interleaved stimuli are possible. This limits the number of points that can be stimulated at the same time. Furthermore, even though cascading multiple switches is possible, given losses of ∼2  dB at each stage, the maximum practical number of stimulation locations is probably about 100 before the device becomes prohibitively bulky and expensive. A method for quick and precise placement of multiple fibers on the cortical surface, such as a perforated matrix, typically used in systematic electrophysiological mapping, also needs to be developed. Another issue is that at 200  μm the minimum spot size is still relatively large. Moreover, the fibers are not entirely transparent, which is a major limitation in combining large arrays with imaging methods since large areas of the brain become blocked from view. In our subsequent designs, we have tried to partially alleviate this problem by leaving space between the fibers, at the expense of reducing the density of stimulation patterns. Coupling into smaller optical fibers is possible but would entail large power losses. For higher channel count, methods such as spatial phase modulation or a galvo mirror-based approach should be considered, using a single powerful diode (coupled to a single mode fiber) rather than a diode stack. A vertical-cavity surface-emitting laser (VCSEL)-based array is another future possibility.^33^

One of the current trends in neuroscience is to combine multiple imaging and stimulation tools to gain a more comprehensive picture of neuronal circuitry. Electrophysiology, optogenetics, and various types of imaging all have their respective advantages and disadvantages. We suggest that INS, with its ability to exploit intrinsic properties of neural tissues, high spatial and temporal precision, and compatibility with other modalities, makes a worthy addition to the neuroscientist’s toolkit.

## Code, data, and materials availability

The data used in the manuscript are available upon reasonable request to the corresponding author.
