# Supplementary figures and images for: Fiberoptic array for multiple channel infrared neural stimulation of the brain
Source: Neurophotonics. 2021 Apr 22;8(2):025005. doi: 10.1117/1.NPh.8.2.025005 (PMC8062107; doi:10.1117/1.NPh.8.2.025005)

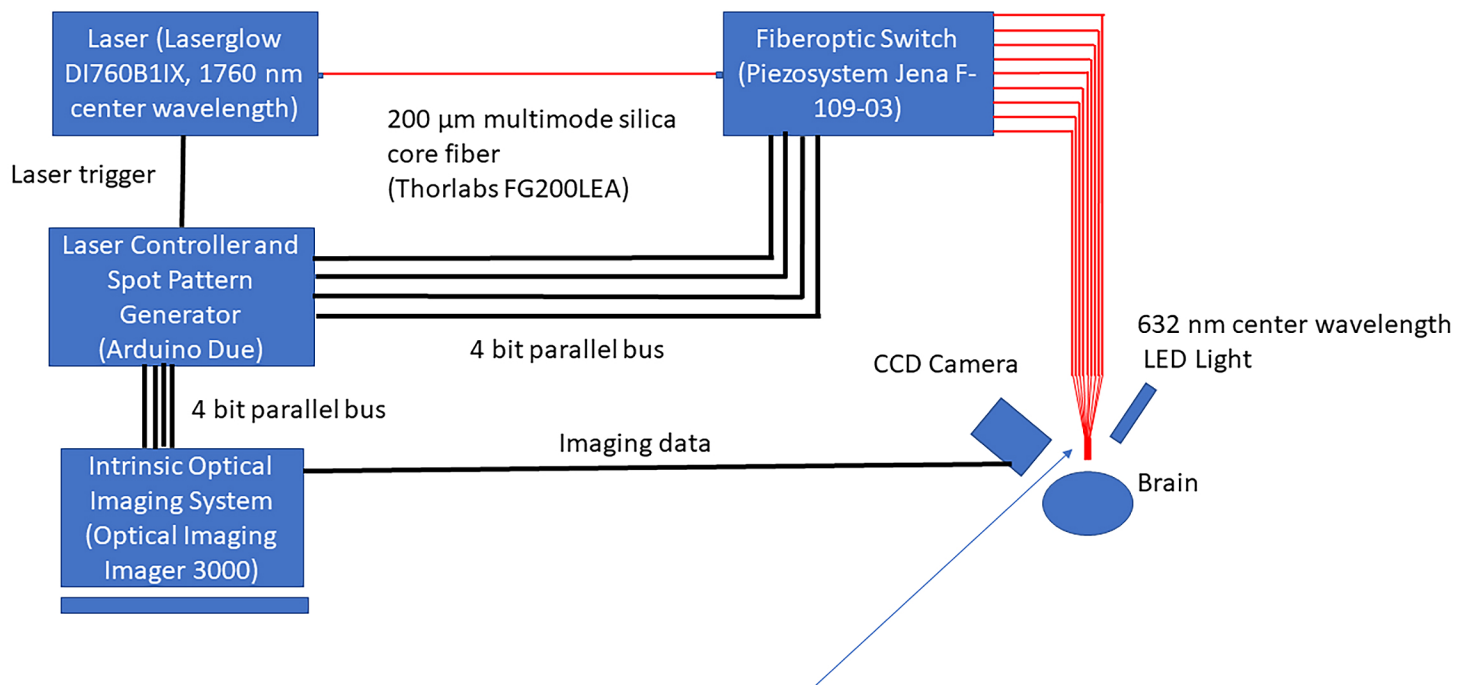

Array consists of bare 240 mm OD silica fibers epoxied in a flat line to a glass coverslip.

Supplement: Supplementary file 1 [file NPh_008_025005_SD001.pdf]
